# Investigation and analysis of training injury and its psychological effects on firefighters in Beijing A cross-sectional study

**DOI:** 10.1097/MD.0000000000035179

**Published:** 2023-09-22

**Authors:** Cheng Gong, Wentao Gao, Bo Zhang, Haifeng Tang, Ying Xie

**Affiliations:** a Department of Rehabilitation Medicine, Beijing Friendship Hospital, Capital Medical University, Beijing, China; b Combat Training Service, Beijing Dongcheng District Fire and Rescue Detachment, Beijing, China; c School of Sport Medicine and Rehabilitation, Beijing Sport University, Beijing, China.

**Keywords:** firefighters, subjective index of well-being, training injuries

## Abstract

Firefighters’ high-intensity training often leads to injuries in the musculoskeletal system. Studies have found that these injuries in the musculoskeletal system may contribute to poor psychological issues. At the same time, low psychological well-being increases the risk of injuries, illness, and mortality. According to research reports, firefighters generally have a good psychological state. So this study aims to survey and analyze the training-related injuries and psychological states of firefighting and rescue personnel in Beijing. This cross-sectional study employed a questionnaire survey to gather data from a total of 214 firefighters in a certain city. The participants were required to complete a questionnaire about musculoskeletal injuries and psychological status, and then these data were statistically analyzed. The incidence of training-related injuries is relatively high among firefighting and rescue teams, with the highest proportions observed in the lower back, knees, and ankles. Overweight and obese firefighters are more prone to ankle injuries. In the group with injuries, the subjective well-being index is lower compared to the group without injuries. Firefighters experiencing moderate to severe pain due to injuries exhibit lower subjective well-being indices compared to those with mild pain. Psychological resilience and the impact of pain on training and sleep can predict the subjective well-being index of firefighters. It is recommended that firefighting and rescue teams enhance preventive measures for musculoskeletal injuries during training to elevate the subjective well-being of firefighters.

## 1. Introduction

Firefighting and rescue teams shoulder vital responsibilities in preventing and resolving major safety risks and responding to various disaster incidents.^[1]^ In the processes of firefighting and rescue operations, firefighters often perform physically demanding tasks while wearing heavy equipment, such as climbing stairs and carrying injured or trapped individuals.^[2]^ Consequently, firefighting and rescue work demands a high level of physical health, fitness, and psychological well-being. To meet the specific physical requirements and skills associated with this profession, firefighters in China must continuously undergo extensive training, including physical fitness training (e.g., strength and endurance) and skills training (e.g., climbing with ropes).^[3]^

As a result, data indicates that firefighters have the highest injury rates among all professions.^[4]^ Musculoskeletal disorders (MSDs) are the most common type of injuries among firefighters, accounting for the vast majority.^[4]^ Furthermore, approximately 55% of musculoskeletal disorders occur during firefighter training rather than at rescue scenes.^[4]^ The incidence of musculoskeletal injuries during firefighting training ranges from 9% to 74%.^[5]^ The most common types of injuries are sprains and strains, primarily affecting the lower back and lower limbs, although specific locations are still debated.^[5]^ Studies have found that 77% of training injuries occur in the lower limbs, with ankles and legs accounting for 32% and 29%, respectively.^[6]^ Ankle sprains and strains were reported to occur most frequently (30.2%), followed by knee injuries (22%), back injuries (18%), and shoulder injuries (31.8%).^[2]^ Other research has found that the highest incidence of training-related MSDs occurs in the back (32%), followed by the knees (23%).^[4]^

The severity of these musculoskeletal injuries has led to increased absenteeism among firefighters. Data shows that the time firefighters take to return to work after musculoskeletal injuries is twice that of private sector workers.^[7]^ Moreover, these injuries result in increased socio-economic costs, with medical expenses related to firefighter musculoskeletal injuries reaching $57,106.^[5]^

Research has revealed that MSDs, particularly chronic ones, can lead to pain, functional impairments, and psychological issues such as anxiety and depression.^[8–11]^Athletes with sports injuries have been found to experience negative psychological effects, and elderly patients with arthritis are more prone to anxiety, depression, and lower levels of well-being.^[12]^ Chronic joint pain patients have shown a decline in mental health status, and the severity of chronic pain is significantly correlated with mental health.^[13]^ Trauma patients in orthopedics have a depression rate as high as 45%, 5 times that of the general population.^[8]^

Furthermore, it has been established that poor psychological states can further impact disease recovery and prognosis, affecting work and life in a vicious cycle.^[8]^ Psychological health is increasingly defined not only by the absence of disease but also by high levels of subjective well-being. Research has shown that reduced subjective well-being is associated with increased depression rates, increase of sports injury risks, prevalence of certain chronic diseases, functional disabilities, and mortality.^[14–17]^ Study reveals that subjective well-being the previous week predicted injury and injury severity the subsequent week in adolescent elite athletes.^[18]^ Other studies have explored the relationship between well-being and lifespan, revealing significant correlations at an individual level.^[14,19]^

Given the high demands on physical and psychological health within the firefighting community, subjective well-being holds significant importance.^[20]^ Cummins proposed a steady-state theory of subjective well-being, suggesting that it is maintained through stable forces such as adaptation, positive emotions, and cognitive buffers, including self-esteem, controllability (the belief that one can achieve desired outcomes through their actions), and optimism (the belief in a positive future regardless of current circumstances).^[21,22]^ However, external pressures may be strong enough to diminish these buffers, leading to a decrease in subjective well-being.^[23]^

Therefore, this study aims to survey and analyze the training-related injuries and psychological states of firefighting and rescue personnel in Beijing. By comparing the subjective well-being differences between personnel with and without injuries, the study aims to observe whether MSDs caused by training affect subjective well-being. The study will also investigate the relationship between pain severity, duration, and subjective well-being, as well as explore how psychological resilience and the impact of pain on daily life, training, emotions, relationships, and sleep contribute to subjective well-being. Additionally, the study will examine whether psychological resilience and the effects of pain can be used to predict the well-being index. Finally, the study will observe whether BMI is a risk factor for training-related injuries, further contributing to injury prevention strategies for firefighters.

## 2. Materials and methods

### 2.1. Study design and research subjects

This is a cross-sectional study. A total of 230 randomly selected fire and rescue personnel from various districts in Beijing participated in the study. The participants were required to complete an online questionnaire within 1 day.

*Inclusion criteria*: male firefighters and rescue personnel aged 20 to 40, without underlying diseases, no musculoskeletal disorders prior to becoming firefighters, good physical health, and voluntary participation in the study.

*Exclusion criteria*: individuals unable to complete the entire questionnaire due to personal reasons, the presence of underlying diseases, and preexisting musculoskeletal disorders before joining the firefighting profession.

### 2.2. Methods

The questionnaire was designed based on relevant literature on training-related injuries among fire and rescue personnel, combined with expert interviews. The questionnaire covered the following aspects:

Primary indicators included 2 aspects: training-related injuries causing pain and functional impairments: Presence of training-related injuries; visual analog scale (VAS) for pain assessment of training-related injuries; Duration of pain caused by training-related injuries. Additionally, based on the Brief Pain Inventory and considering the firefighting context, the impact of pain on daily life, training, emotions, relationships, and sleep was assessed using a 0 to 10 scale, where higher scores indicated the greater impact of pain.^[24]^ Subjective Index of Well-being (IWB) and Connor-Davidson Resilience Scale (CD-RISC): Subjective well-being was assessed using the IWB questionnaire, comprising 8 items for overall emotional well-being and 1 item for life satisfaction, each scored on a scale of 1 to 7. The SWB index ranged from 2.1 to 14.7, with higher scores indicating stronger subjective well-being. The Psychological Resilience was assessed using the CD-RISC included 25 items, with scores ranging from 0 to 4, indicating “never,” “rarely,” “sometimes,” “often,” and “almost always.” The total score ranged from 0 to 100, with higher scores indicating better psychological resilience.^[25,26]^

Secondary indicators included 4 aspects: general information: gender, age, height, weight, body mass index (BMI), years of work, and years of training. Daily training: total daily training hours, training types, training frequency, and the rest day distribution. Training and occurrence of training-related injuries: presence or history of training-related injuries; site and type of injuries. Measures taken after injuries: actions taken after injuries.

The questionnaire was filled out anonymously, and participants provided informed consent before participating. This study was approved by the Ethics Committee of Beijing Friendship Hospital, Capital Medical University (ethics number: 2022-P2-320-01).

### 2.3. Data analysis

Data were analyzed using SPSS 20.0 software (IBM Corporation, New York, NY). Descriptive statistics were used for continuous variables, independent sample *t* tests for comparisons between 2 groups, analysis of variance for comparisons among multiple groups, and multiple linear regression analysis for predictive modeling. The comparison of disease risk between 2 groups is conducted using an odds ratio. A significance level of *P* < .05 was considered statistically significant.

## 3. Result

### 3.1. General information

A total of 230 survey questionnaires were distributed to fire and rescue personnel, resulting in 214 valid responses. Among the 214 respondents, all were male, with an average age of 29.36 ± 2.65 years, average height of 173.48 ± 12.80 cm, average weight of 78.50 ± 21.65 kg, average BMI of 24.10 ± 2.51, average years of work of 9.64 ± 3.74 years, and average years of training of 9.23 ± 3.84 years. The average daily training duration was 6.26 ± 1.98 hours. The longest-serving firefighter had worked for 17 years, with 17 years of training experience. The firefighter with the longest training duration trained for 10 hours per day, and all firefighters trained for 5 days and rested for 2 days each week. The average IWB for fire and rescue personnel was 12.42 ± 2.64, and the average CD-RISC score was 71.68 ± 21.70.

Among the respondents, 112 had training-related injuries, with an average age of 29.22 ± 2.56 years, average BMI of 23.92 ± 2.55, average years of work of 10.04 ± 3.52 years, and average daily training duration of 6.24 ± 1.86 hours. 34 (30.36%) injured firefighters continued training despite their injuries.

There were 102 respondents without training-related injuries, with an average age of 29.52 ± 2.75 years, average BMI of 24.30 ± 2.47, average years of work of 9.19 ± 3.94, and average daily training duration of 6.27 ± 2.12 hours. Basic characteristics showed no significant differences between the 2 groups (*P* > .5) (Table 1).

**Table 1:** Comparison of basic information on firefighters with and without training injuries.

	Firefighters with training injury (n = 112)	Firefighters without training injury (n = 102)	*P* value
Age (yr)	29.22 ± 2.56	29.52 ± 2.75	.415
BMI	23.92 ± 2.55	24.30 ± 2.47	.263
Year of working experience (yr)	10.04 ± 3.52	9.19 ± 3.94	.096
Daily training time (h)	6.24 ± 1.86	6.27 ± 2.12	.902

Values are presented as mean ± SD.

BMI = body mass index.

*P < .05.

**P < .01.

### 3.2. Comparison of the subjective IWB firefighters with and without injury

**Figure 1.**
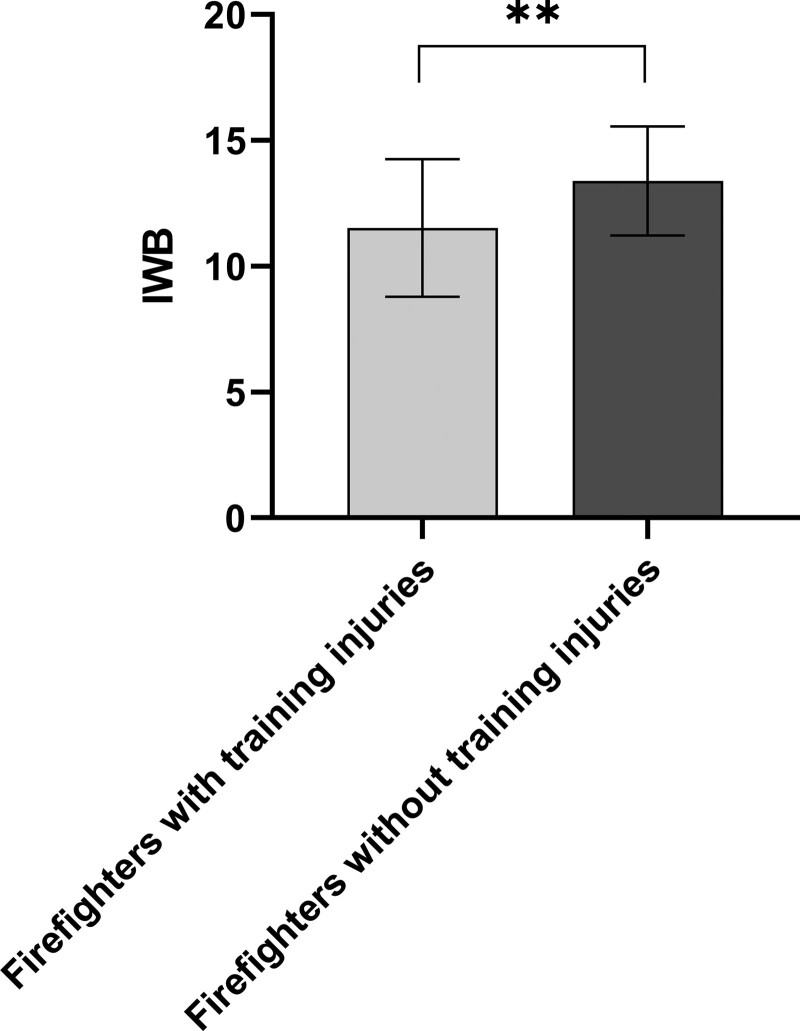
Comparison of IWB for firefighters with and without training injuries. Values are presented as mean ± SD. IWB = index of well-being. *P < .05 and **P < .01.

The average subjective well-being index scores for firefighters with and without injuries were 11.52 ± 2.73 and 13.39 ± 2.16, respectively. The subjective well-being index of the group with injuries was significantly lower than that of the group without injuries (*P* < .01) (Fig. 1).

### 3.3. Comparison of subjective IWB among firefighters with different levels of pain due to training injuries

**Figure 2.**
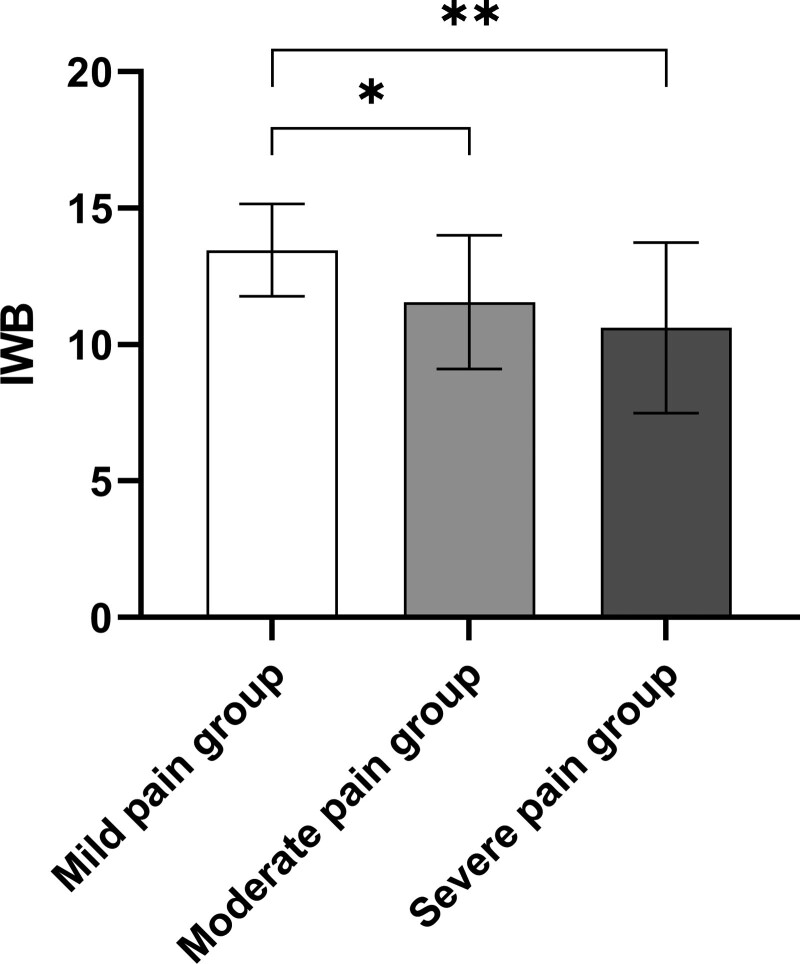
The effect of different pain levels on IWB. Values are presented as mean ± SD. IWB = index of well-being. *P < .05 and **P < .01.

Using Welch’s analysis of variance, 3 groups were formed based on the severity of pain caused by training injuries: mild pain group (VAS: 1–3) with 16 participants, moderate pain group (VAS: 4–6) with 60 participants, and severe pain group (VAS: 7–9) with 36 participants. The average impact of pain on IWB scores for these groups were as follows: mild pain group, 13.46 ± 1.69; moderate pain group, 11.55 ± 2.45; severe pain group, 10.62 ± 3.12. There was a significant difference in IWB scores among the different pain severity groups (Welch *F* = 10.261, *P* = .0002). Post hoc analysis revealed that the average IWB score decreased significantly from the mild pain group to the moderate pain group (difference: −1.91, 95% confidence interval [CI]: 0.17–3.65, *P* = .028), and from the mild pain group to the severe pain group (difference: −2.84, 95% CI: 0.98–4.70, *P* = .001). However, the difference in IWB score from the moderate pain group to the severe pain group was not statistically significant (difference: −0.93, 95% CI: −0.53 to 2.40, *P* = .281) (Table 2, Fig. 2).

**Table 2:** The effect of different pain levels on IWB.

Group	IWB
Mild pain group (n = 16)	13.46 ± 1.69
Moderate pain group (n = 60)	11.55 ± 2.45
Severe pain group (n = 36)	10.62 ± 3.12**
*F* value	10.261
*P* value	.0002

Values are presented as mean ± SD.

IWB = index of well-being.

*P < .05.

**P < .01.

### 3.4. Comparison of subjective IWB among firefighters with different duration of pain

This study used independent sample *t* test to compare the average IWB scores between firefighters with acute and subacute pain (pain duration less than 3 months) and those with chronic pain (pain duration more than 3 months). The results showed that the average IWB scores were 11.35 ± 2.56 for firefighters with acute and subacute pain and 11.82 ± 3.02 for those with chronic pain. There was no significant difference in IWB scores between the 2 groups (*P* = .381) (Table 3).

**Table 3:** The effect of different pain duration on IWB.

	Acute and subacute pain (n = 71)	Chonic pain (n = 41)	*P* value
IWB	11.35 ± 2.56	11.82 ± 3.02	.381

Values are presented as mean ± SD.

IWB = index of well-being.

*P < .05.

**P < .01.

### 3.5. Multiple linear regression predictive model for subjective IWB

**Figure 3.**
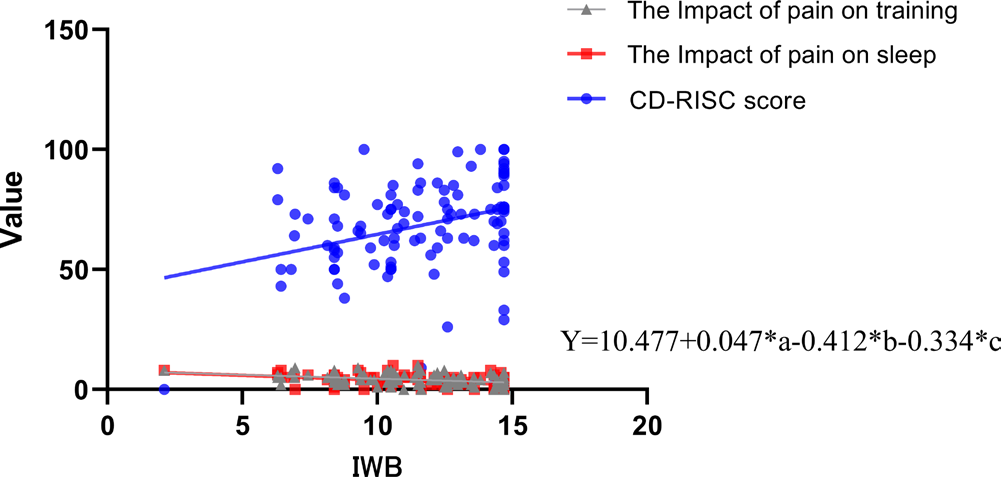
Multiple linear regression prediction model for the IWB. Values are presented as mean ± SD. a = CD-RISC score, b = the impact of pain on training, c = the impact of pain on sleep, CD-RISC = Connor-Davidson resilience scale, IWB = index of well-being, Y = IWB. *P < .05 and **P < .01.

The results of this study showed that the average IWB of the group with injuries was 11.52 ± 2.73. The average scores on the psychological resilience scale, the impact of pain on daily life, training, emotions, relationships with others, and sleep were 68.14 ± 19.71, 3.64 ± 2.19, 4.04 ± 2.32, 3.73 ± 2.28, 2.71 ± 2.43, and 3.43 ± 2.42, respectively. A multiple linear regression model was used to predict the IWB based on the scores of psychological resilience, the impact of pain on life, training, emotions, relationships with others, and sleep. The regression model was statistically significant (*F* = 8.972, *P* < .001, adjusted *R*^2^ = 0.301). It was found that 3 independent variables included in the model—psychological resilience, the impact of pain on training, and the impact of pain on sleep—had a statistically significant impact on the IWB (*P* < .05). The regression equation is detailed in Figure 3.

### 3.6. Proportion of firefighters with lower limb injuries based on different BMI

The results of this study showed that the most common injured areas among firefighters due to training were the waist (59 cases, 52.68%), followed by the knee (49 cases, 43.75%), and then the ankle (33 cases, 29.46%). Among firefighters with injuries, 49 were overweight or obese (BMI>=24), and 63 were normal or underweight (BMI < 24). Compared with firefighters with BMI < 24, those with BMI>=24 had a higher likelihood of ankle injuries, with an odds ratio of 3.188 (95% CI: 1.368–7.427). The risk of lumbar spine (odds ratio = 1.028, 95% CI: 0.486–2.171) and knee joint (odds ratio = 0.599, 95% CI: 0.280–1.285) injuries did not show significant differences (Table 4).

**Table 4:** Risk of lower extremity injuries in firefighters with different BMI.

	Odds ration for BMI (BMI>=24/BMI < 24)	95% CI
Firefighters with lumbar injury	1.028	0.486–2.171
Firefighters with knee injury	0.599	0.280–1.285
Firefighters with ankle injury	3.188	1.368–7.427

BMI = body mass index, CI = confidence interval.

## 4. Discussion

The purpose of this study is to compare the differences in subjective well-being between firefighters with and without MSDs, in order to observe whether MSDs resulting from training affect psychological states. By observing the differences in subjective well-being among firefighters with varying levels and durations of pain, the relationship between pain and well-being is explored. The study also examines the impact of pain on psychological resilience, daily life, daily training, emotions, interpersonal relationships, and sleep, aiming to identify the factors through which training injuries affect the well-being index and establish a regression prediction model for well-being. Finally, the study examines whether BMI is a risk factor for training injuries, thereby providing a basis for injury prevention.

The results of this study reveal that firefighters with MSDs caused by training experience a significant decrease in subjective well-being compared to those without MSDs. This finding is consistent with previous research that has shown a decline in psychological states among patient populations with orthopedic trauma, surgery, chronic back pain, and chronic joint pain.^[8,12,13]^ The results suggest a connection between the psychological state of firefighters and MSDs caused by training. Cummins theory of the stable model of subjective well-being posits that an individual’s well-being remains within a certain range, and intense emotions triggered by external stimuli can cause well-being to deviate from this range. The stability mechanism is activated to regulate subjective well-being and bring it back to the set range. Generally, subjective well-being is in a state of dynamic stability, but if negative experiences exceed the body’s adaptive capacity, it can lead to a decrease in well-being and a greater deviation from the normal set range, making it less likely to rebound to the equilibrium level.^[21]^ Therefore, the possible reason for the appearance of these results is that the negative effects of pain and functional impairment caused by MSDs surpass the firefighters” adaptive capacity, resulting in a negative impact on their subjective well-being.

Furthermore, the study finds that compared to the mild pain group, the moderate to severe pain group experiences a significant decrease in subjective well-being. This suggests a correlation between the intensity of pain and well-being scores, supporting the majority of research findings. Most previous studies have shown a significant negative correlation between the intensity of pain and psychological states, while a small number of studies have found no direct or indirect correlation.^[27–29]^ The difference in these results may be related to whether pain causes pain interference. Studies by Iddon et al have found that the impact of pain, rather than its intensity, drives the relationship between chronic pain and decreased psychological states. When the pain intensity is high but does not interfere with important aspects of daily life, such as activities, work, and relationships, patients are more likely to maintain a positive mindset. However, when pain affects normal life, work, and social participation, its impact on psychological well-being is greater.^[30]^ In recent years, the understanding of pain has shifted from a primarily biomedical model to a biopsychosocial approach, indicating that the influence of pain on psychological well-being is multidimensional, multifaceted, and complex.^[27]^ Although there is no significant difference in well-being index between firefighters with severe pain and those with moderate pain, there is a trend of decreased well-being, which may be attributed to the small sample size. The results of this study suggest that, for firefighters, prevention of training injuries and timely treatment of existing injuries are crucial to avoid exacerbating pain and having a greater impact on their psychological states.

In addition to pain duration, the study also compares the impact of disease duration on the well-being index. The results show no significant difference in subjective well-being between the chronic pain group and the acute or subacute pain group, indicating that the duration of pain is not significantly correlated with the well-being index among firefighters. This finding aligns with the results of Dysvik et al’s study.^[10]^ Although there is limited research comparing the impact of acute or subacute pain and chronic pain on psychological states, numerous studies have confirmed that chronic pain can lead to adverse psychological states such as anxiety and depression.^[9]^ The mechanisms underlying chronic pain include central sensitization, impaired descending pain inhibition pathways, and immunological mechanisms.^[31]^ Studies have shown that the mechanisms leading to psychological issues in individuals with chronic pain are related to the cognitive and behavioral changes caused by pain, with fear-avoidance behavior being a common pattern among chronic back pain patients.^[9,31]^ Avoidance perpetuates or amplifies fear, which can lower pain thresholds, leading to further disability and impairment.^[31]^ The lack of a decrease in subjective well-being among firefighters with chronic pain in this study may be attributed to their better psychological states and adaptive coping abilities. The data from this study also indicates that approximately 30.36% of injured firefighters continued training despite their injuries, which may indicate a reduction in fear-avoidance behavior caused by pain among firefighters.

Additionally, this study establishes a multiple linear regression prediction model, finding that psychological resilience, the degree of pain’s impact on training, and the impact of pain on sleep significantly influence the well-being index, supporting previous research. The theory of personal goal satisfaction suggests that when an individual perceives obstacles to valuable activities and future outcomes, suffering increases.^[30]^ Cuff et al^[32]^ found that pain interference has a greater impact on depression symptoms than pain intensity alone. It can be inferred that for firefighters, who spend most of their time training during the week, sleep is crucial for physical recovery and fatigue reduction. Therefore, training and sleep are of utmost importance for firefighters. When MSDs resulting from training affect firefighters, the impact on their IWB performance is maximized if both training and sleep are affected. Psychological resilience refers to an individual’s ability to adapt and adjust to adversity, trauma, tragedy, threats, or other significant stressors, and is a psychological quality.^[33]^ This aligns with Cummins’ theory of stable well-being, which emphasizes the concept of “adaptability.” Prior research indicates that athletes’ psychological resilience can decrease the sports injury risks.^[33]^ Thus, psychological resilience, the impact of pain on training and sleep, significantly influence the well-being index.

Furthermore, the study finds that the most frequently injured parts of firefighters due to training are the lumbar region, knee, and ankle, consistent with the results of previous studies. This is possibly due to the fact that in China, firefighters engage in many training activities simulating real-life scenarios, such as carrying mannequins, gas cylinders, and breathing apparatuses, climbing stairs with heavy loads, and extensive running, which can easily lead to injuries in the lower back and lower limbs. The study also reveals that among firefighters with training-related injuries, those who are overweight or obese are more likely to experience ankle injuries than those with normal or lean body weights. Previous research has confirmed a correlation between BMI and the risk of sports injuries.^[34]^ Studies have shown that about 56% of firefighters are overweight or obese, and firefighters with a BMI over 30 have a threefold higher injury rate than those with a BMI below 30.^[35]^ It is also suggested that higher body mass index is a significant predictor of ankle injuries in male athletes.^[35]^ The potential reason for this result is that individuals with higher body weights experience greater torque and pressure on joints, and they also require stronger muscle strength. As a result, they are more prone to ankle injuries. This result suggests the importance of weight management for firefighters to prevent the risk of ankle injuries.

## 5. Limitations

This study has certain limitations. Firstly, the sample size is relatively small. Secondly, the study only explores the relationship between training-induced musculoskeletal injuries and subjective well-being, but lacks an in-depth investigation into the interaction between these factors and the reasons behind the occurrence of training injuries. Therefore, future research should aim to increase the sample size and further explore the causes of training injuries and the mutual influence between injuries and psychological states.

## 6. Conclusion

In conclusion, training injuries are common among firefighting and rescue teams, with injuries primarily occurring in the lumbar region and lower limbs. Overweight and obese firefighters are more susceptible to ankle injuries. Firefighters with injuries experience a lower subjective well-being compared to those without injuries. Among firefighters, those with moderate to severe pain due to injuries have a lower subjective well-being compared to those with mild pain. Psychological resilience, the impact of pain on training, and the impact of pain on sleep are predictive factors for the well-being index. It is recommended to strengthen the prevention of musculoskeletal injuries during training to enhance the subjective well-being of firefighters.

## Author contributions

**Conceptualization:** Ying Xie.

**Data curation:** Wentao Gao, Bo Zhang, Haifeng Tang.

**Formal analysis:** Cheng Gong.

**Funding acquisition:** Cheng Gong.

**Methodology:** Ying Xie.

**Supervision:** Haifeng Tang.

**Writing – original draft:** Cheng Gong.

**Writing – review & editing:** Ying Xie.
